# Fabrication of High Temperature Oxidation Resistance Nanocomposite Coatings on PEO Treated TC21 Alloy

**DOI:** 10.3390/ma13010011

**Published:** 2019-12-18

**Authors:** Kai Zhou, Faqin Xie, Xiangqing Wu, Shaoqing Wang

**Affiliations:** School of Aeronautics, Northwestern Polytechnical University, Xi’an 710072, China; zhouk785@163.com (K.Z.); wxqwsy@nwpu.edu.cn (X.W.); wsq1209@mail.nwpu.edu.cn (S.W.)

**Keywords:** plasma electrolytic oxidation, nanocomposite coatings, TC21 titanium alloy, ZrO_2_ nanoparticles, high temperature oxidation resistance

## Abstract

The effects of ZrO_2_ nanoparticles in a NaAlO_2_ electrolyte on the thickness, morphology, composition, structure, and high temperature oxidation resistance of plasma electrolytic oxidation (PEO) coatings on a TC21 titanium alloy were investigated. The coating thickness increased with increasing concentration of ZrO_2_ nanoparticles in the electrolyte, accompanied by a decrease in the porosity of the coating surface. The PEO coatings formed in the ZrO_2_ nanoparticle-free electrolyte were composed of Al_2_TiO_5_. ZrTiO_4_, m-ZrO_2_, and t-ZrO_2_ were detected in the PEO coatings produced by the electrolyte that contained ZrO_2_ nanoparticles, which indicated that the deposition mechanism of the nanoparticles was partly reactive incorporation. The high temperature oxidation resistance of the TC21 titanium alloy at 650 °C and 750 °C was improved by 3–5 times after PEO treatment. The oxidation mechanism involved oxygen diffusing inward to form an oxide layer at the interface of the PEO coating and substrate.

## 1. Introduction

Titanium alloys are attractive for applications in the aerospace industry because of their high strength to weight ratio, excellent corrosion resistance, great composite compatibility, and remarkable fatigue properties [1,2,3]. TC21 titanium alloys are a damage-tolerant type of titanium alloy that has been used in aircraft landing gears, connecting pieces, engine frameworks, and engine cabin partitions that have temperature requirements [4,5]. However, its widespread application in high temperature parts of the aircraft are still limited due to poor high temperature oxidation resistance. To date, various coating methods have been studied to improve the high temperature oxidation resistance, including electron-beam physical vapor deposition [6], plasma spraying [7], hot dipping [8], laser cladding [9], arc ion plating [10], magnetron sputtering [11], sol-gel [12], and plasma electrolytic oxidation (PEO) [13].

PEO is a process to produce ceramic coatings on the metal surface that have corrosion and wear resistance, and desirable electrical and thermal properties [14]. Initial studies of PEO mostly focused on the effects of different electrolyte compositions and electrical parameters on the properties of the coatings. However, the porous microstructure is detrimental to the corrosion resistance and high temperature oxidation resistance of the PEO coating. Therefore, increasing research on PEO composite coatings has been carried out recently. One strategy is to prepare composite coatings by combining PEO with other surface treatment technologies [15,16,17,18,19]. However, this secondary composite technology undoubtedly increases the workload and energy consumption. Another strategy is to prepare nanocomposite coatings by adding various insoluble nanoparticles into the electrolyte. In this process, the type and concentration of nanoparticles directly affects the properties of the coatings. The most commonly added particles are various oxides including TiO_2_ [20], Al_2_O_3_ [21], ZrO_2_ [22], SiO_2_ [23], CeO_2_ [24], and La_2_O_3_ [25]. The particles can achieve either reactive or inert incorporation into the coating while imparting excellent properties to the coating.

ZrO_2_ has the advantages of strong thermal shock resistance, high temperature resistance, and good chemical stability. Many studies have prepared composite coatings by adding ZrO_2_ nanoparticles to the electrolyte, but most of them focused on the wear and corrosion resistance of the coatings [26,27,28,29]. Very limited studies have been done on the high temperature oxidation properties of this kind of composite coating.

In this paper, three different concentrations of ZrO_2_ nanoparticles were added to the electrolyte to study changes in the thickness, surface morphology, composition, and structure of the coatings. The reactive or inert incorporation mechanism of the ZrO_2_ nanoparticles was determined. The oxidation behavior and mechanism of the composite coatings at different temperatures were studied.

## 2. Materials and Methods

### 2.1. Materials

Rectangular specimens (30 mm × 20 mm × 2 mm) of two-phase TC21 titanium alloy were used as substrates for PEO. The nominal composition of this alloy by wt.% is 6.23 Al, 2.15 Zr, 2.10 Sn, 2.93 Mo, 1.60 Cr, 2.03 Nb, and balance Ti. Prior to the coating process, the samples were abraded using 240, 400, 800, 1000 grits SiC emery papers in succession, the substrates were then ultrasonically cleaned in ethanol and then dried in air.

### 2.2. Coating Preparation

**Figure 1:**
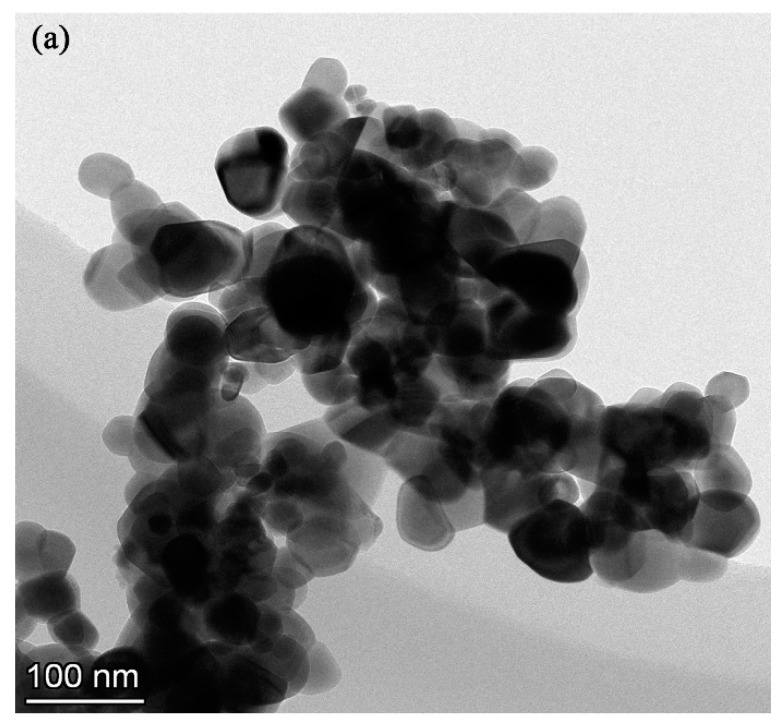

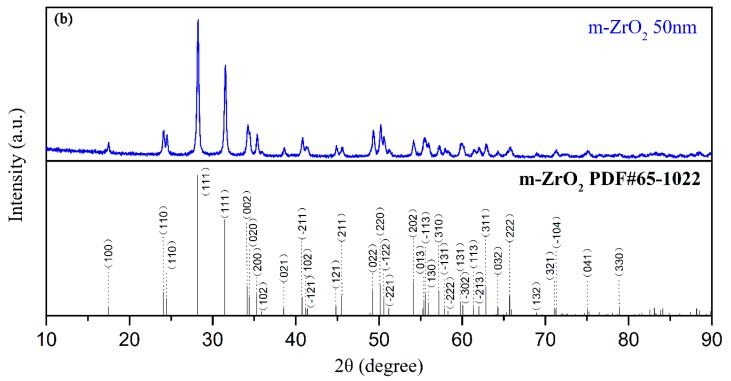
(**a**) TEM image, (**b**) XRD patterns of m-ZrO_2_ nanopowder.

The electrolyte for the PEO process was composed of sodium aluminate, sodium phosphate, sodium hydroxide, and different concentrations of monoclinic-ZrO_2_ (m-ZrO_2_) nanoparticles with an average size of 50 nm (Figure 1). The detailed chemical concentration of the electrolyte is listed in Table 1.

A pulsed unipolar power supply unit was used to carry out the PEO process. A 304 stainless steel double-walled chamber containing the electrolyte was used as a cathode through which cooled water was pumped to keep the electrolyte temperature close to 20 °C. A TC21 titanium alloy rectangular sample immersed in the electrolyte was used as the anode. During the PEO process, the electrical parameters were fixed as follows: anodic current density of 10 A/dm^2^, frequency of 600 Hz, duty cycle of 10% and treatment time of 20 min. To ensure the uniform dispersion of m-ZrO_2_ nanoparticles in the electrolyte, the corresponding amount of m-ZrO_2_ nanoparticles was added to the prepared 2 L volume of electrolyte. After continuous stirring for 24 h, the electrolyte was ultrasonicated for 30 min and transferred to the 304 stainless steel chamber to start the PEO process. During the treatment, the electrolyte was stirred continuously. After PEO treatment, the coating samples were rinsed in deionized water and dried in air.

### 2.3. High Temperature Oxidation Tests

The TC21 titanium alloys and coating samples were put into an alumina crucible and placed in a muffle furnace for cyclic oxidation experiments. Before the cyclic oxidation experiments, the samples pre-dried at 150 °C were weighed together with the alumina crucibles, which were pre-dried to a constant mass at 800 °C. The cyclic oxidation experiment temperatures were 650 °C and 750 °C. The total oxidation time was 100 h. Every 10 h, the experimental samples were taken out and placed into a dryer. After cooling in static air, the mass of each sample was weighed by a Sartorius BSA 124S analytical balance (accuracy of 10^−4^ g).

### 2.4. Coating Characterization

The surface and cross-sectional morphology and elemental compositions of the PEO coatings before and after high temperature oxidation were observed by scanning electron microscopy (SEM, JSM-6460, JEOL, Tokyo, Japan) equipped with energy dispersion X-ray spectrometry (EDS, Oxford INCA, Oxford, UK). The phase composition was investigated by X-ray diffraction (XRD), using a D8 Bruker Advantage instrument (Bruker, Karlsruhe, Germany) with a step size of 0.05° and a scan range from 10° to 90° (in 2θ). Transmission electron microscopy (TEM) characterization with EDS was performed by using a FEI Talos F200X (FEI, Hillsboro, OR, USA). The TEM specimen preparation was as follows: pieces of the coating were scraped off and ground into a powder; the powder was dispersed in ethanol and ultrasonicated for 20 min; and then, several microliters of the solution was added dropwise onto a carbon-coated copper grid for subsequent TEM analysis. The zeta potentials of ZrO_2_ nanoparticles in the electrolyte were measured using a zeta potential analyzer (Nano zs; Malvern Panalytical, Malvern, UK).

## 3. Results

### 3.1. Surface SEM Analysis

**Figure 2:**
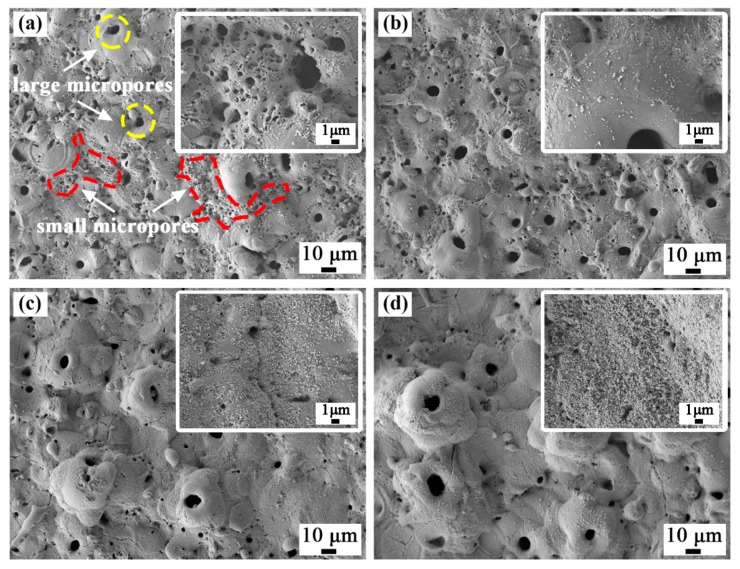
Surface morphology of the plasma electrolytic oxidation (PEO) coatings formed in (**a**) bath A, (**b**) bath B, (**c**) bath C, and (**d**) bath D.

The scanning electron micrographs of the surface of the coated samples are shown in Figure 2. The coatings prepared in four different electrolytes had a typical volcanic cone morphology, and there were different size discharge micropores in the middle and around the volcanic cones. For the coatings prepared in bath A, there were two kinds of discharge micropores on the surface. One type comprised small discharge micropores continuously distributed in the red labelled area with a diameter less than 2 μm. The other type comprised the independent discharge micropores marked in the yellow area with a large aperture of 5 to 8 μm. When the ZrO_2_ nanoparticles were added to the electrolyte, the number of fine discharge micropores decreased as the concentration of the nanoparticles increased. For the coating prepared in bath D, the fine discharge micropores almost disappeared. This was because with an increase in the concentration of the ZrO_2_ nanoparticles in the electrolyte, the thickness of the coating increased, which led to an increase in the discharge intensity. Furthermore, from the enlarged image in Figure 2, it can be observed that the fine white ZrO_2_ nanoparticles deposited on the surface of the coatings, which played an obvious sealing effect, and the deposition amount increased with an increase in the concentration of ZrO_2_ nanoparticles. Table 2 shows the porosity of the coatings determined with by ImageJ software, and the porosity of the composite coatings decreased obviously.

Table 2 shows the EDS surface scan results for each coating surface. As the concentration of ZrO_2_ nanoparticles in the electrolyte increased, the content of aluminum and titanium in the coatings gradually decreased, and the content of zirconium increased. This also indicated that deposition of the ZrO_2_ nanoparticles occurred.

### 3.2. Cross-Sectional SEM Analysis

**Figure 3:**
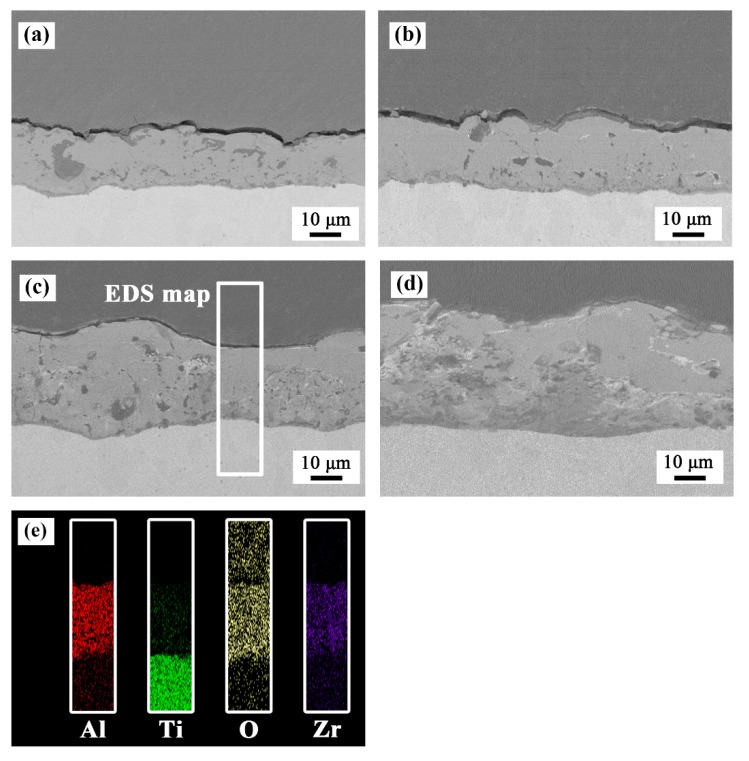
Cross-sectional morphology of the PEO coatings formed in (**a**) bath A, (**b**) bath B, (**c**) bath C, and (**d**) bath D. (**e**) EDS elemental mapping of the sample formed in bath C.

As shown in Figure 3, all the coated samples showed good adhesion to the substrate without any discontinuity at the interface. The results of elemental mapping showed that Al, Ti, O, and Zr elements were uniformly distributed in the coatings. Table 2 shows the thickness increased with an increase in the concentration of ZrO_2_ nanoparticles.

### 3.3. Phase Composition

**Figure 4:**
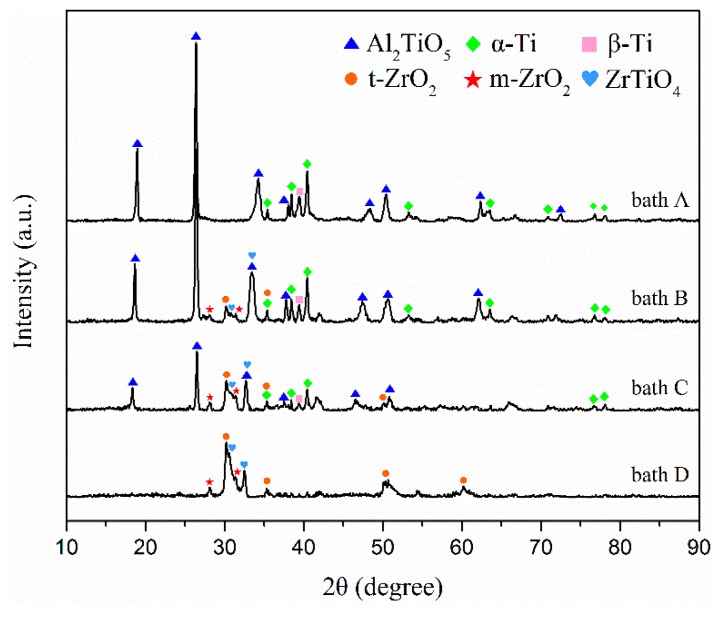
XRD patterns of the PEO coatings formed in baths A, B, C, and D.

The X-ray diffraction peaks of the coatings formed in baths A, B, C, and D are shown in Figure 4. The coated samples prepared in bath A consisted mainly of Al_2_TiO_5_. Alpha and beta titanium peaks in the TC21 alloy spectra can still be observed because of the relatively low atomic number, thin thickness, and high porosity of the coated samples.

The m-ZrO_2_, tetragonal-ZrO_2_ (t-ZrO_2_), and ZrTiO_4_ can be observed in the composite coatings prepared in baths B, C, and D. At the same time, it can be observed that the alpha and beta titanium peaks for TC21 alloy in the spectra decreased gradually. One reason was the composite coating contained zirconium, which has a large atomic number. Another reason was that as the ZrO_2_ concentration increased, the coating thickness increased and the porosity decreased. Titanium peaks were not observed in the coatings prepared in bath D. In addition, with an increase in the concentration of ZrO_2_ nanoparticles in the electrolyte, the peak strength of Al_2_TiO_5_ decreased gradually, which also indicated that ZrO_2_ nanoparticles were deposited on the coating surface.

### 3.4. TEM Analysis

**Figure 5:**
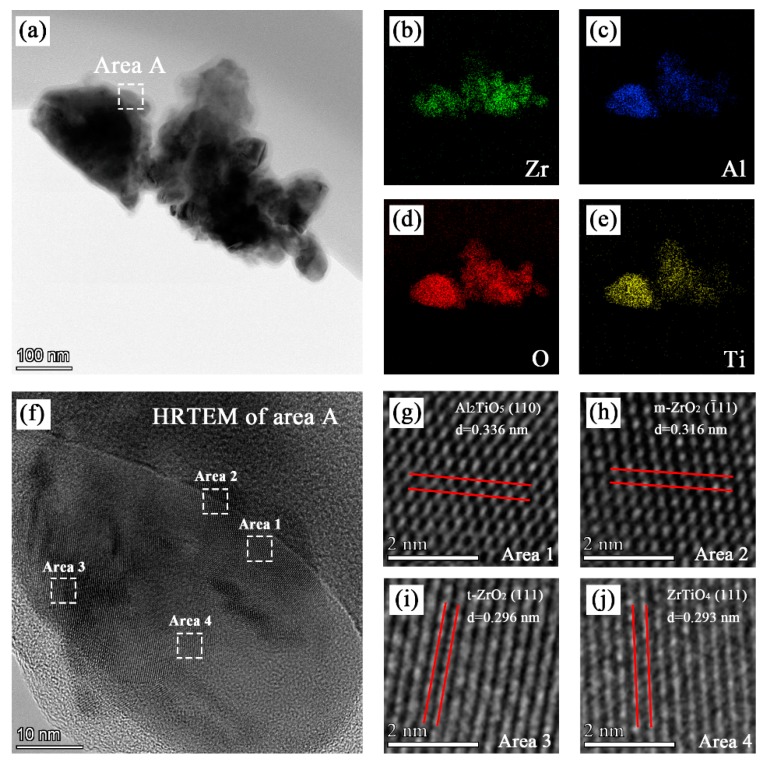
TEM, EDS, and HRTEM analysis of the PEO coating formed in bath C: (**a**) TEM images; (**b**), (**c**), (**d**), and (**e**) EDS mapping of the distribution of zirconium, aluminum, oxygen, and titanium, respectively; and (**f**), (**g**), (**h**), (**i**), and (**j**) HRTEM images of certain regions.

The TEM image of pieces scraped off the coatings formed in bath C is shown in Figure 5a. The EDS maps showed in Figure 5b–e indicate that the coating contained zirconium, aluminum, oxygen, and titanium. As shown in Figure 5f–j, various lattice fringes can be observed in the HRTEM image of area A. The crystal plane spacing obtained from the lattice fringes in area 1, area 2, area 3, and area 4 were 0.336 nm, 0.316 nm, 0.297 nm, and 0.293 nm, respectively, which correspond to the (110) crystal plane of Al_2_TiO_5_, (−111) crystal plane of m-ZrO_2_, (111) crystal plane of t-ZrO_2_, and (111) crystal plane of ZrTiO_4_, respectively.

### 3.5. Oxidation Kinetics

**Figure 6:**
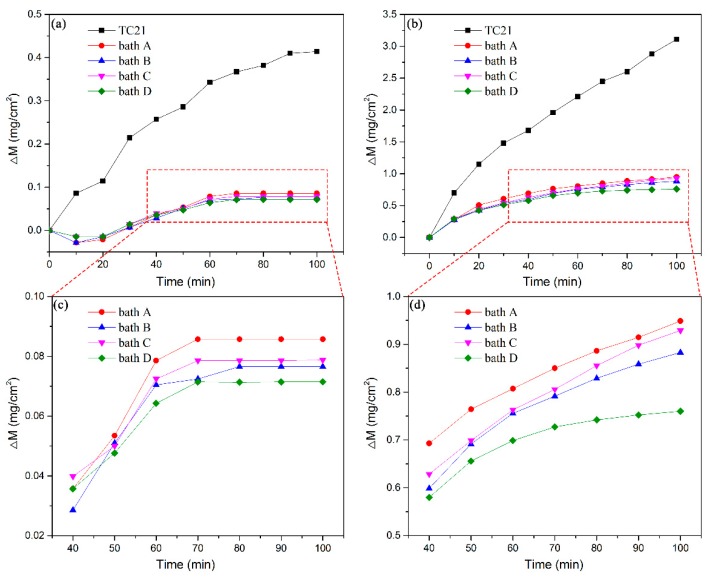
Oxidation kinetic curves of TC21 alloys and the PEO coatings oxidized at different temperatures: (**a**) and (**c**) 650 °C and (**b**) and (**d**) 750 °C.

Figure 6 depicts the oxidation kinetics curves of the uncoated and coated TC21 alloys after cyclic oxidation at 650 °C and 750 °C. The weights of the four kinds of coatings decreased at the beginning of cyclic oxidation at 650 °C. Literature reports indicate that the coatings contain unstable hydrated compounds or carbonates, which decompose during the initial stage of high temperature oxidation, resulting in a decrease in the weight [30]. After 20 h of high temperature oxidation, the weight of the four coatings began to increase with the oxidation time. However, after several cycles, the coating quality was almost unchanged from 60 h of oxidation time and onward. The weight of the TC21 alloys continued to increase rapidly during the first 60 h of the oxidation experiment. Although there was still obvious weight gain after 60 h, the growth rate gradually slowed. Finally, after 100 h of oxidation, the weight change of the TC21 alloys was 0.41 mg/cm^2^, and the weight change of coated samples prepared in baths A, B, C, and D was 0.086 mg/cm^2^, 0.077 mg/cm^2^, 0.079 mg/cm^2^, and 0.071 mg/cm^2^, respectively, which was nearly five times lower than that of TC21 alloys.

During the cyclic oxidation experiment at 750 °C, the slopes of the curves for the TC21 alloys were very large during the first 20 h. After 20 h, the slopes decreased slightly, but the weights still increased almost linearly. The weight change was 3.11 mg/cm^2^ after 100 h of oxidation. The oxidation kinetic curves for the four kinds of coatings were pseudo-parabolic. The weight gain of the composite coatings prepared in baths B, C, and D were 0.88 mg/cm^2^, 0.93 mg/cm^2^, and 0.76 mg/cm^2^ respectively, which were slightly lower than that of the coatings prepared in bath A, which was 0.95 mg/cm^2^.

The high temperature cyclic oxidation experiments at both temperatures showed that the PEO coatings can significantly improve the high temperature oxidation resistance of TC21 alloys. The high temperature oxidation resistance of the ZrO_2_ composite coatings was slightly better than that of the conventional coatings.

### 3.6. SEM/XRD Analysis of the PEO Coatings after Cyclic Oxidation

**Figure 7:**
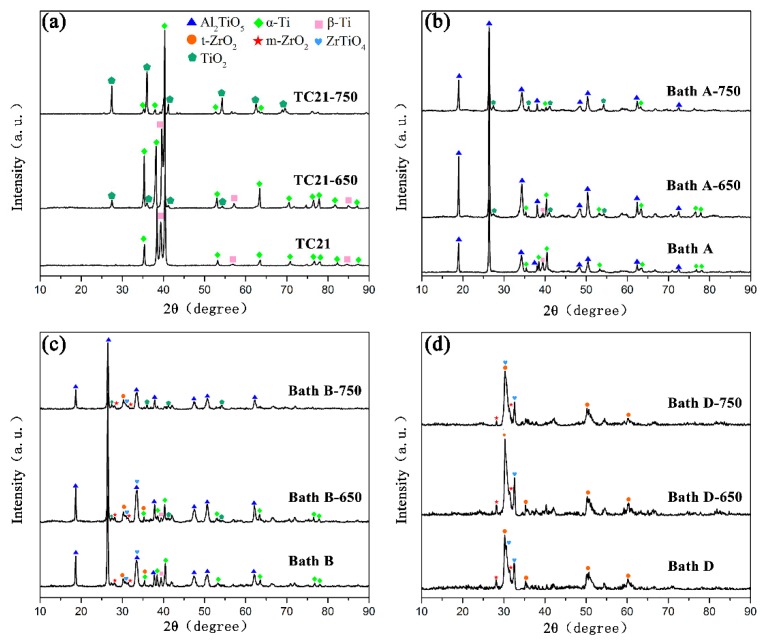
XRD patterns of (**a**) TC21 alloys and the PEO coatings formed in (**b**) bath A, (**c**) bath B, and (**d**) bath D after high temperature oxidation tests.

Figure 7 shows the XRD spectra of TC21 alloys and PEO coatings after high temperature cyclic oxidation at 650 °C and 750 °C. (The results of coatings produced in bath B and bath C were similar. Figure 7 gives only the results of the coating produced in bath B). 

Rutile can be observed for the TC21 alloys because the transition temperature from anatase to rutile is 600 °C ~ 1000 °C. With the increase of oxidation temperature, the intensity of the rutile peaks increased and the peak intensities of α-Ti and β-Ti decreased.

For coatings produced in baths A, B, and C, the phenomenon was similar to that for the TC21 alloys after high temperature oxidation. Rutile was found in all these coatings, but no Al_2_O_3_ peak was observed, which indicated that the Al_2_TiO_5_ phase in the coatings was stable and did not decompose during high temperature oxidation. The rutile came from the oxidation of the titanium matrix.

Although weight gain occurred, rutile was not observed in the coatings produced in bath D after high temperature oxidation. This was because the coatings themselves were thick, and the oxidation weight gain was very small, indicating that the formation of rutile was relatively small.

**Figure 8:**
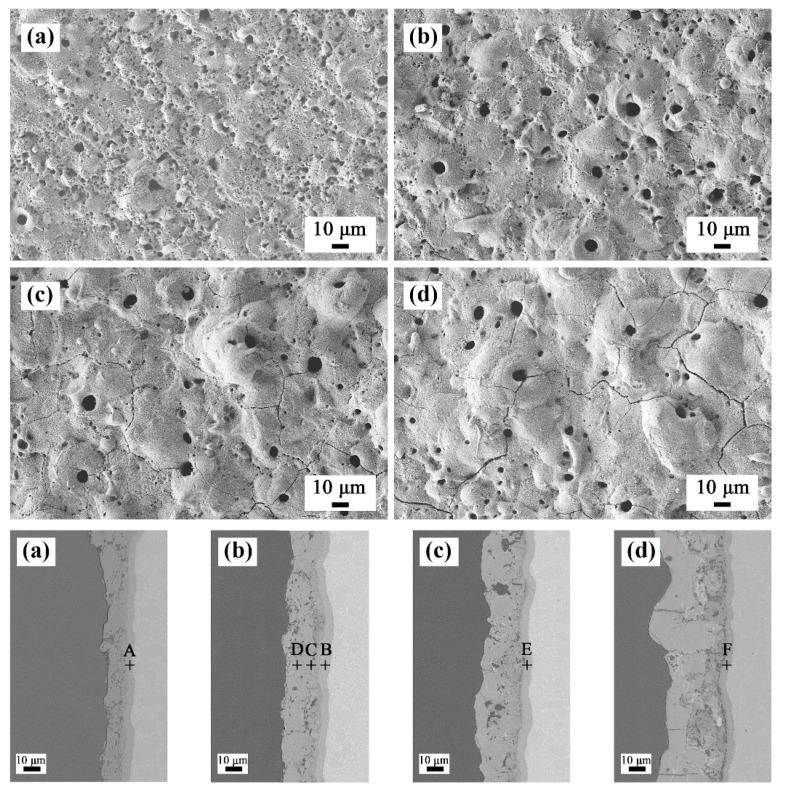
Surface and cross-sectional morphologies of the PEO coatings after 750 °C oxidation tests: (**a**) bath A, (**b**) bath B, (**c**) bath C, and (**d**) bath D.

Figure 8 shows the surface and cross-sectional microstructures of the PEO coatings after oxidation at 750 °C. Some distinct cracks appeared on the surface of the coatings. The cracks in the coatings prepared in baths A and B were relatively few in number, while number of the cracks in the coatings prepared in baths C and D were obviously increased. From the cross-sectional morphology, it can be seen that the porosity increased slightly compared with that before oxidation. Vertical cracks can be observed in sample D. In addition, an interlayer of approximately 4 μm in thickness was formed at the interface between the TC21 alloys and coatings. The composition of the interlayers in atom percentage was as follows: point A (Ti 42.8%, O 56.6%, and Al 0.6%), point B (Ti 42.6%, O 56.6%, Al 0.4%, and Zr 0.4%), point E (Ti 39.1%, O 59.8%, Al 0.7%, and Zr 0.4%), and point F (Ti 41.3%, O 57.2%, Al 0.5%, and Zr 0.5%). The results showed that an oxide layer formed at the interface. However, in addition to rutile, there should be enrichment of titanium atoms in the interlayer. In addition, the low aluminum content meant that a small amount of alumina was produced during the oxidation process, which resulted in no corresponding peak on the XRD spectrum.

The above results were because the formation free energy and equilibrium oxygen pressure of Al/Al_2_O_3_ and Ti/TiO_2_ are similar, and the formation rate of TiO_2_ and Al_2_O_3_ is almost the same at the beginning of oxidation. However, the growth activation energy of TiO_2_ are significantly lower than that of Al_2_O_3_, and the formation rate of TiO_2_ was faster in subsequent stages [31]. Moreover, the composition of point C in atom percentage was (Ti 9.4%, O 57.0%, Al 28.5%, and Zr 5.1%) and that of point D was (Ti 9.7%, O 56.1%, Al 29.0%, and Zr 5.2%), which showed that the coatings themselves did not change during the oxidation process.

## 4. Discussion

### 4.1. Nanoparticle Deposition Mechanism

The deposition mechanism of the nanoparticles in the PEO coatings is determined by many factors, such as the substrate, electrolyte, and electrical parameters. The ZrO_2_ nanoparticles in this experiment were found to achieve partly reactive incorporation, resulting in the inclusion of m-ZrO_2_, t-ZrO_2_, and ZrTiO_4_ in the coatings. The zeta potential of the m-ZrO_2_ nanoparticles was −30 mV in alkaline electrolytes. They migrated to the anode and deposited on the coating surface inertly during the electrophoresis and mechanical stirring processes. In addition, the molten oxide ejected from the discharge micropores also enclosed the m-ZrO_2_ nanoparticles near the anode. The active deposition of m-ZrO_2_ nanoparticles included two aspects. First, the m-ZrO_2_ underwent phase transformation to form t-ZrO_2_. Second, ZrO_2_ nanoparticles that reacted with melted TiO_2_ formed during the PEO process and formed ZrTiO_4_. The m-ZrO_2_ transformed to t-ZrO_2_ at 1200 °C and t-ZrO_2_ transformed to c-ZrO_2_ at 2370 °C [32]. The formation temperature of ZrTiO_4_ ranged from 1100 °C ~ 1200 °C [33]. In summary, the reaction temperature of the experimental process should be between 1373 °C and 2370 °C.

### 4.2. High Temperature Oxidation Behavior and Mechanism

**Figure 9:**
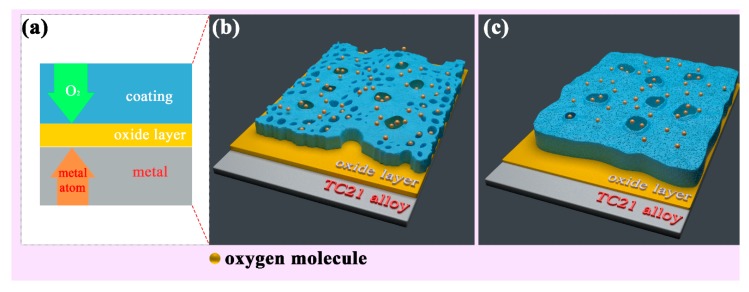
two-dimension (**a**) and three-dimension schematic diagram of the high temperature oxidation mechanism of the PEO coatings formed (**b**) without ZrO2 and (**c**) with ZrO2.

The essence of high temperature oxidation is that metal ions and oxygen molecules diffuse through the oxide coating and react when they meet. As shown in Figure 9a, the oxidation mechanism in this experiment was that the oxygen molecules diffuse inward, and preferentially reacted with the titanium atoms to form a layer of rutile at the interface between the coatings and TC21 alloys. The oxidation kinetics curves show that the PEO coatings substantially hindered the diffusion of the oxygen molecules. The oxidation weight gain of the coated samples was significantly smaller than that of the TC21 alloys. The protective property of the coatings was also better than that of the oxide spontaneously formed during the oxidation process of the TC21 alloys at high temperature. However, the discharge micropores on the surface of the coating inevitably became the diffusion channel for the oxygen molecules. With increasing oxidation temperature, the diffusion rate of the oxygen molecules through the coating increased, and the reaction rate of the metal/oxide interface increased, resulting in an increased oxidation reaction.

Figure 9b,c show that the surface porosity of conventional PEO coatings was relatively large, and the oxygen molecule diffusion channel increased accordingly. The coating was relatively thin and the resistance to oxygen diffusion was comparatively weak. For the composite coatings, the thickness of the coating was significantly increased, and the ability to block oxygen molecules was increased. The porosity of the coating was significantly reduced due to the deposition of the nanoparticles on the surface of the coating and the barrier effect on the oxygen molecules was further enhanced. In addition, the composite coatings contained m-ZrO_2_, t-ZrO_2_, and ZrTiO_4_, which are stable at high temperatures, have a low thermal expansion coefficient, and do not readily delaminate and decompose at high temperatures [34]. Therefore, the high temperature oxidation resistance of the composite coatings was better than that of the conventional coatings.

## 5. Conclusions

Ceramic composite coatings were formed on TC21 alloys by plasma electrolytic oxidation using a m-ZrO_2_ nanoparticle suspension as the electrolyte. The high temperature oxidation behavior and mechanism at 650°C and 750°C were investigated. The following conclusions can be drawn:

1. The composite coatings were mainly composed of m-ZrO_2_, t-ZrO_2_, and ZrTiO_4_, indicating that ZrO_2_ nanoparticles achieved partial reactive incorporation.
2. As the concentration of ZrO_2_ nanoparticles in the electrolyte increased, the thickness of the composite coating increased and the porosity decreased.
3. The high temperature oxidation resistance of TC21 alloys was improved by 3–5 times after PEO treatment, and that of the composite coatings were improved by nearly 20% compared with that of the conventional coatings.

## Figures and Tables

**Table 1:** The detailed compositions of electrolyte.

Sample	NaAlO_2_ (g·L^−1^)	Na_3_PO_4_ (g·L^−1^)	NaOH (g·L^−1^)	m-ZrO_2_ (g·L^−1^)
bath A	15	4	1.5	0
bath B	15	4	1.5	2
bath C	15	4	1.5	4
bath D	15	4	1.5	8

**Table 2:** Area energy dispersion X-ray spectrometry (EDS) analysis, thickness, and porosity of the PEO coatings.

Sample	Area EDS Analysis (at. %)				Thickness (μm)	Porosity (%)
	O	Al	Ti	Zr		
bath A	58.4	30.7	10.9	/	21.5	5.7
bath B	58.0	27.3	9.3	5.4	28.4	4.8
bath C	57.4	23.9	9.2	9.5	34.1	3.1
bath D	56.6	17.8	8.5	17.1	39.3	3.3
